# 3D-printed bioactive scaffolds from nanosilicates and PEOT/PBT for bone tissue engineering

**DOI:** 10.1093/rb/rby024

**Published:** 2018-12-15

**Authors:** James K Carrow, Andrea Di Luca, Alireza Dolatshahi-Pirouz, Lorenzo Moroni, Akhilesh K Gaharwar

**Affiliations:** 1 Department of Biomedical Engineering, Texas A&M University, College Station, TX, USA; 2 Tissue Regeneration Department, MIRA Institute for Biomedical Technology and Technical Medicine, University of Twente, Enschede, The Netherlands; 3 DTU Nanotech, Center for Intestinal Absorption and Transport of Biopharmaceuticals, Technical University of Denmark, 2800 Kgs, Lyngby, Denmark; 4 Department of Complex Tissue Regeneration, MERLN Institute for Technology-Inspired Regenerative Medicine, Maastricht University, Universiteitsingel 40, Maastricht, The Netherlands; 5 Department of Materials Science, Texas A&M University, College Station, TX, USA and; 6 Center for Remote Health Technologies and Systems, Texas A&M University, College Station, TX, USA

**Keywords:** 3D printing, nanocomposites, two dimensional (2D) nanoparticles, copolymer, tissue engineering

## Abstract

Additive manufacturing (AM) has shown promise in designing 3D scaffold for regenerative medicine. However, many synthetic biomaterials used for AM are bioinert. Here, we report synthesis of bioactive nanocomposites from a poly(ethylene oxide terephthalate) (PEOT)/poly(butylene terephthalate) (PBT) (PEOT/PBT) copolymer and 2D nanosilicates for fabricating 3D scaffolds for bone tissue engineering. PEOT/PBT have been shown to support calcification and bone bonding ability *in vivo*, while 2D nanosilicates induce osteogenic differentiation of human mesenchymal stem cells (hMSCs) in absence of osteoinductive agents. The effect of nanosilicates addition to PEOT/PBT on structural, mechanical and biological properties is investigated. Specifically, the addition of nanosilicate to PEOT/PBT improves the stability of nanocomposites in physiological conditions, as nanosilicate suppressed the degradation rate of copolymer. However, no significant increase in the mechanical stiffness of scaffold due to the addition of nanosilicates is observed. The addition of nanosilicates to PEOT/PBT improves the bioactive properties of AM nanocomposites as demonstrated *in vitro.* hMSCs readily proliferated on the scaffolds containing nanosilicates and resulted in significant upregulation of osteo-related proteins and production of mineralized matrix. The synergistic ability of nanosilicates and PEOT/PBT can be utilized for designing bioactive scaffolds for bone tissue engineering.

## Introduction

Additive manufacturing (AM) has shown promise in regenerative medicine though fabrication of 3D-printed scaffolds to support and enhance tissue regeneration and function [1–3]. A range of AM techniques such as stereolithography, fused deposition modeling, and laser sintering have produced cost-effective and high-resolution systems to fabricate complex structures for various biomedical applications. Scaffolds are made in a layer-by-layer manner that enables the direct construction of complex structures with very high precision. AM aligns particularly well with building patient-specific implants as use of medical imaging and computer-aided design can be combined to obtain custom designed tissue implants.

Extrusion-based AM is extensively used for bone tissue engineering due to its ability to produce various biomaterials including thermoplastics [4–10]. However, tradeoffs between mechanical strength, bioactivity, printability and biological characteristics have resulted in limited choice of synthetic polymers for AM. Thermoplastic polymers, such as poly (ɛ-caprolactone) (PCL), poly(lactic acid), polyether ether ketone, are investigated for printing 3D bone grafts [4–10]. Co-polymers such poly(ethylene oxide terephthalate) (PEOT)/poly(butylene terephthalate) (PBT) (PEOT/PBT), also known as Polyactive, are also investigated for AM [11–13] due to its bone bonding ability [14–20]. However, these polymeric biomaterials provide often a bioinert substrate as opposed to biologically active materials [21–27], and lack ability to direct bone formation.

A range of bioactive nanomaterials are incorporated in synthetic polymers to direct cell functions including hydroxyapatite nanoparticles [28, 29], graphene [30, 31] and synthetic nanosilicates [32, 33]. Recently, nanosilicates demonstrated high cytocompatibility, as well as effective bioactivity to induce differentiation of human mesenchymal stromal cells (hMSCs) into osteogenic lineages in absence of growth factors [32, 33]. It has been shown that hMSCs treated with nanosilicates upregulate osteogenic markers and protein production such as alkaline phosphatase (ALP), osteocalcin, osteopontin and induce mineralization [33–35]. The nanosilicates-based scaffolds are also highly biocompatible and result in minimum immune response under *in vivo* conditions [36, 37]. Furthermore, nanosilicates can be homogenously distributed within a polymeric matrix due to their charged surfaces [38–41]. This capability provides an advantage over alternative bioactive nanomaterials, like hydroxyapatite or mesoporous bioactive glasses, which require chemical modifications to the surface to endow the particles with improved electrostatic characteristics [42, 43].

Here, we report additive manufactured bioactive 3D scaffolds from PEOT/PBT and nanosilicates for bone tissue engineering. We investigated the effect of nanosilicate addition to PEOT/PBT on structural, mechanical and biological properties. Specifically, the printability, physiological stability and cellular interactions of fabricated scaffolds are evaluated. The biological activity of hMSCs on 3D scaffolds are determined using *in vitro* experiments. It is expected that these nanostructured bioactive scaffolds can be used for non-load bearing bone tissue engineering.

## Experimental

### Materials

PEOT/PBT copolymer was obtained from PolyVation B.V. (Groningen, The Netherlands). The composition used in this study was 1000PEOT70PBT30, as synthesized earlier [16, 44]. The initial PEG block molecular weight was 1000 g/mol for co-polymerization through a melt polycondensation reaction. Specifically, the reaction resulted in the oxidation of PEG which is subsequently terepthalated. The weight ratios between PEOT and PBT blocks following copolymerization were 70 and 30%, respectively. Nanosilicates (Laponite XLG), obtained from BYK Additives and Instruments, was placed in an oven at 100°C for 4 h to remove environmental water from the hygroscopic nanoparticles.

### Fabrication of PEOT/PBT 3D-printed scaffolds

A 10 g of PEOT/PBT along with nanosilicates at 5, 10 or 15 wt.% were dissolved into dimethylformamide (DMF) and allowed to mix overnight. A 5 and 10 wt.% of nanosilicate solution could be successfully extruded from the printer. To confirm the presence of nanosilicates energy-dispersive x-ray spectroscopy (EDS) analysis was used. Due to the low volatility of the solvent, the pre-polymer solution was cast into a petri dish and allowed to further evaporate for 12 h until the solution qualitatively increased in viscosity. Subsequently, the nanocomposite was placed into 100% ethanol for 2 days to enable DMF exchange with the more volatile solvent. Ethanol was added at an equal volume as the original DMF. This exchange further increased the qualitative viscosity of the composite, rendering the material with sufficient printable capabilities, as determined on a binary scale (i.e. printable fibers or not printable). Scaffolds were fabricated with an extrusion-based AM system (SysENG GmbH, Germany) [45]. The nanocomposite was loaded into a syringe and extruded through a needle with an inner diameter of 400 µm at a pressure between four and five bars. The spacing between extruded fibers was set to 1.2 mm, whereas the thickness of each layer was set to 500 µm. A woodpile configuration was selected based on previous AM studies targeting bone engineering [46–48]. Following printing of layered structures, scaffolds of uniform size could be acquired through punches or cutting with a scalpel.

### Physical characterization

Nanosilicate size was confirmed using transmission electron microscopy (TEM). Specifically, images were captured with a JEOL-JEM 2010 microscope with an accelerating voltage of 200 kV on a carbon grid. Both pure PEOT/PBT and PEOT/PBT/Nanosilicates compositions were used to determine physical properties. Polymer compositions were generated *via* dissolution of the polymer in DMF at a concentration of 5 wt.%. Nanocomposites contained nanosilicates at a concentration of 5–15 wt.% per weight of polymer added. The pre-polymer solution was vortexed for 1 min followed by heating at 40°C for 20 min, and this process was repeated until the polymer was completely dissolved. Upon dissolution, the solution was cast into a petri dish and left under vacuum until the solvent had evaporated, leaving a thin polymer film. The film was removed from the dish and was biopsy punched into a variety of shapes for subsequent experiments. Films were utilized over printed scaffolds to ensure measured effects were independent of potential variations in scaffold architectures following extrusion. After film fabrication, interactions of the polymer system within a variety of aqueous environments were evaluated. Accelerated degradation of both compositions occurred *via* monitoring dry weight of polymer strips submerged in 0.01 M NaOH for various time points. Scaffolds were likewise placed under the same degradative conditions for 24 h and examined under electron microscopy. Scanning electron microscopy (SEM) images were collected on a JEOL NeoScope microscope on gold sputter coated samples (gold thickness ∼25 nm). Separate EDS analysis was performed on alternate samples assisted by EDAX Inc (Mahwah, NJ, USA). To evaluate hydrophilicity and its effect on protein adhesion, films were first biopsy punched into 6-mm circles. Static contact angles were measured with an Attension CAM 200 optical tensiometer (Biolin Scientific AB, Stockholm, Sweden) after 15 s of applying a 5 µl droplet of water. Protein adhesion was quantified using fluorescent bovine serum albumin (BSA). Briefly, a solution of protein (100 µg/ml) was pipetted over the surface of films adhered onto the bottom of a 48 well-plate. The well plate was placed on a shaker table for 30 min to distribute the protein over the surface over the films. After shaking, films were washed twice with PBS to remove lightly bound protein and then a detergent solution (2% sodium dodecyl sulfate (SDS)) was incubated over the films for 2 h. The supernatant was subsequently analyzed using a Nanodrop 3300 spectrophotometer (Thermo Scientific, USA) for fluorescence excitation/emission at 470/515 nm.

### Mechanical properties

An Xpert 7600 mechanical tester (ADMET, USA) was utilized for analyzing mechanical strength of 3D-printed scaffolds. Briefly, rapid fabrication scaffolds were tested using uniaxial compression tests, respectively, at a strain rate of 0.1 mm/min for cyclical testing and 0.2 mm/min for single run. The modulus was calculated from the elastic region corresponding to 0.10–0.20 strain, with energy dissipation and percent recovery between cycles calculated from the engineering stress–strain curves. Modulus was normalized between printed samples by accounting for volume occupied by printed strands (i.e. number of fibers per layer, number of layers and fiber diameter) within the overall volume of the construct. Energy dissipated was calculated from the area between the loading and unloading curves during cyclic runs.

### *In vitro* studies

For cell studies, scaffolds were first sterilized for 30 min in 70% ethanol. Scaffolds were then incubated overnight in basal media conditions, which was composed of minimum essential medium (α-MEM) with 16.5% fetal bovine serum and penicillin (100 U/ml) and streptomycin (100 µg/ml). hMSCs (Lonza, USA) were then seeded onto scaffolds (5 × 10^5^ cells/scaffold) using a fibronectin-supplemented media (300 µg/ml fibronectin). After 4 h of incubation at 37°C, additional basal media was added to the scaffolds to sustain proliferation for 2–3 days. Media was carefully exchanged every 2–3 days to ensure attached cells were not washed off of the scaffolds and maintain contact with the biomaterial surface. Cell proliferation was monitored via Alamar Blue assay (Thermo Scientific, USA) following the manufacturer’s protocol. The absorbance of the reduced solution was measured using a microplate reader (Infinite M200PRO, TECAN, Europe) to calculate the percent reduction of resazurin to resorufin, which was then normalized to the control. The presence of extracellular ALP in differentiated cells seeded on 3D printed scaffolds was evaluated using nitro-blue tetrazolium/indolyl phosphate (NBT/BCIP) staining (Thermo Scientific, USA). The scaffolds were washed with PBS and then incubated with 0.25 ml of NBT/BCIP at 37°C for 30 min on Days 7, 14 and 21. Samples were then washed with PBS and quantified and dissolved with 10% acetic acid to quantify activity. The synthesis of mineralized matrix by hMSCs on the nanocomposites was analyzed on Day 21 using Alizarin Red S staining. The cells and matrix on the scaffolds were fixed using 2% glutaraldehyde for 15 min with 0.5% Alizarin Red S (pH 4.2; Sigma-Aldrich, USA) subsequently added for staining. The samples were washed with distilled water to remove unbound Alizarin Red S after a 15-min incubation in which positively stained calcium was dissolved and absorbance quantified on a microplate reader. The concentration of double-stranded DNA (dsDNA) was measured via a PicoGreen dsDNA Quantification Kit (Invitrogen, Molecular Probes, USA) to normalize samples to cell number.

### Statistics

Collected and analyzed data were presented as mean ± SD (*n* = 5). One-way analysis of variance (ANOVA) with Tukey *post-hoc* analysis was utilized to determine statistically significant differences for mechanical properties of the scaffolds, and two-way ANOVA for ALP analysis over multiple time points. Significance for protein adhesion and Calcium deposition at Day 21 were calculated via two-tailed t-test. Significance was indicated as *P < 0.05, **P < 0.01, ***P < 0.001.

## Results and discussion

### Fabrication and characterization of 3D scaffolds

**Figure 1:**
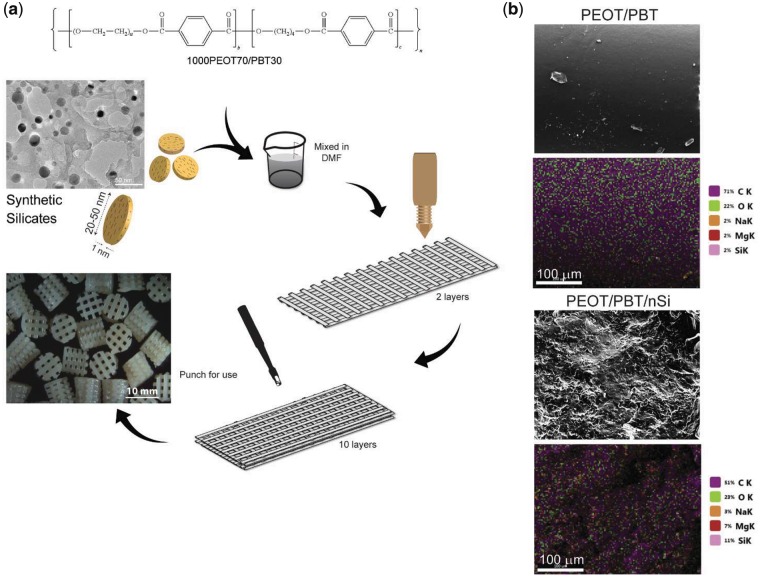
Fabrication of 3D nanocomposite scaffolds. **(a)** The addition of PEOT/PBT and nanosilicates in DMF resulted in a homogeneous solution for extrusion; **(b)** EDS analysis indicated an increase of mineral-specific elements following introduction of nanosilicates throughout the 3D structure

The scaffolds containing PEOT/PBT and nanosilicates were fabricated *via* an extrusion-based AM system (Fig. 1a). Nanosilicates were characterized with TEM which confirmed disc-like shape, with diameters ranging from 20-50 nm. To obtain bioactive nanocomposites, nanosilicates were mixed with PEOT/PBT in DMF in a concentration of 10% wt./wt. nanosilicates to copolymer. Qualitative analysis of composite viscosity determined an optimal nanosilicate concentration to be 10%. Specifically, this ratio of nanosilicate to polymer resulted in continuous and homogeneous flow of the nanocomposite during extrusion. At higher concentrations (i.e. 15% nanosilicate), the solution remained extrudable, however the viscosity increased beyond the capabilities of achieving continuous flow, thereby resulting in scaffold defects. This, in combination with the hypothesis that a 10% nanosilicate solution within the scaffold would stimulate a phenotypic change in cultured cells due to previous studies [49], determined the final nanosilicate concentration. Following mixing of polymer and nanomaterial components, the flow properties of the printing ink were monitored. To improve the flow profile of the solutions, solvent exchange was utilized with ethanol to improve viscosity for more controlled printing. Ethanol was introduced after formation of the composite solution in DMF to enable greater dispersal and homogeneous distribution of nanosilicates. Optimization of printing characteristics correlates with improved resolution and spatial control over the course of many layers. Qualitative observations of the extrudate indicated printable viscosities with minimal clogging at the nozzle or within the syringe barrel. Although many systems rely on thermoplastic characteristics of polymer filaments to achieve layer-to-layer integration and scaffold stability, this system utilized evaporation of solvent to render solidification of extruded fiber. To achieve rapid fabrication, localized air flow around the scaffold during the manufacturing process can expedite solvent evaporation.

To determine the distribution of nanosilicates within the fabricated structure, EDS analysis was performed (Fig. 1b). PEOT/PBT analysis showed the presence of carbon and oxygen from the copolymer backbone. The addition of nanosilicates into the PEOT/PBT matrix resulted in additional peaks for sodium, magnesium and silicon. Uniform distribution of the nanosilicates was also observed throughout the nanocomposites. This would result in uniform physical and bioactive characteristics of the manufactured scaffold. Uniform distribution of nanosilicates within the scaffold might be attributed to high viscosity of the pre-polymer solution and electrostatic repulsion between nanosilicates [50, 51].

**Figure 2:**
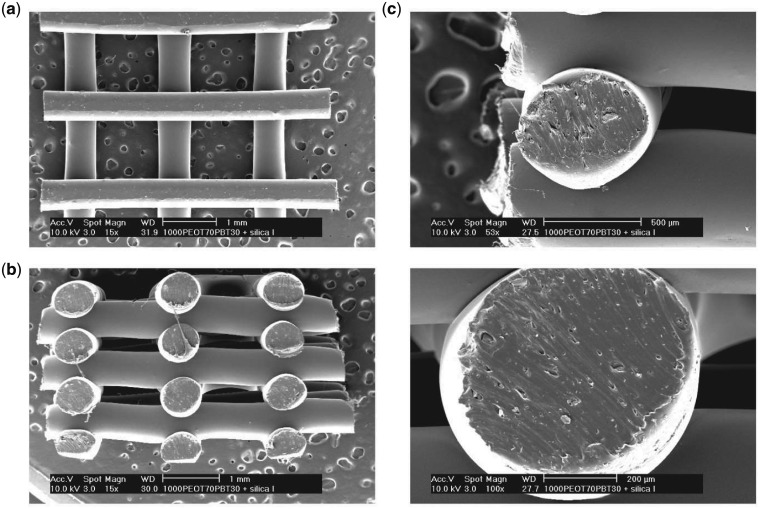
SEM Images of nanocomposite scaffold architecture following extrusion with 400-µm diameter nozzle. **(a)** Top view of single layer print; **(b)** side view of seven-layer print displaying inter-layer spacing and lateral spacing between fibers; **(c)** cross-sectional view of single fibers following biopsy from the macro structure

The addition of nanosilicates did not affect print fidelity of the PEOT/PBT solution. Nanocomposite extrudate followed slightly larger dimensions relative to the inner diameter of the nozzle (400 µm) with fiber width around 0.64 ± 0.04 mm as determined from SEM (Table 1). In addition, the fiber spacing was calculated as 1.17 ± 0.04 mm, whereas the layer thickness as 0.6 ± 0.07 mm. This resulted in a porosity of the scaffolds of 74.4 ± 0.3% as calculated from Moroni *et al.* [9]. Moreover, layer-to-layer interactions were maintained prior to solvent evaporation during the fabrication process, which could be more easily visualized with the woodpile arrangement (Fig. 2). The spacing of the extruded fibers was selected to display structural integrity of fibers, particularly coverage distance with minimal sagging of struts. Furthermore, the woodpile structure enabled the deposited scaffolds to be cut into new sizes as demonstrated by biopsy punching into defined geometries. This also demonstrated the malleability of the manufactured scaffold. Future scaffolds could be designed with slightly narrower fiber spacing to generate tighter pores around 500 µm to further facilitate osteogenic differentiation of seeded stem cells. Additionally, this would improve mechanical properties while still maintaining nutrient diffusion maintaining future bone in-growth [52].

**Table 1:** Printed scaffold measurements

Fiber diameter	0.64 ± 0.04 mm
Layer thickness	0.6 ± 0.07 mm
Fiber spacing (effective)	1.17 ± 0.04 mm

### Effect of nanosilicates on protein adsorption and degradation of 3D scaffolds

**Figure 3:**
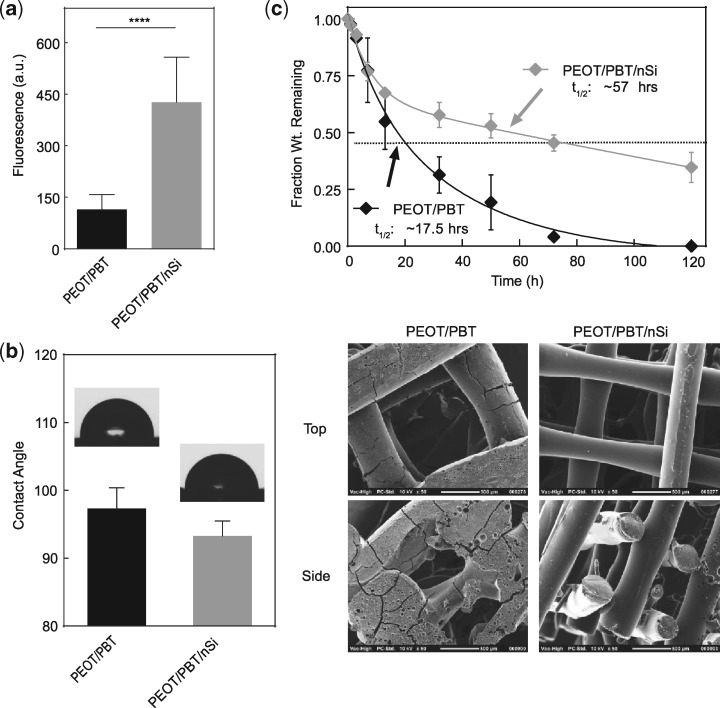
Scaffold responses in physiological conditions. **(a)** The addition of nanosilicates facilitates protein adsorption on 3D scaffolds. **(b)** The addition of nanosilicates did not alter the hydrophobicity of the scaffold material. **(c)** The effect of nanosilicates was evaluated on degradation properties of PEOT/PBT. Pure polymer scaffolds showed enhanced degradation, while addition of nanosilicate slowed the degradation kinetics of scaffold. SEM images scaffold morphology after subjecting to degradation solution (0.01 M NaOH) for 24 h

Adsorption of protein on biomaterials surface facilitate cell adhesion and spreading [53, 54]. Films of scaffold compositions were subjected to solubilized BSA in which the quantity of adsorbed protein was determined after washing. The results showed that significantly greater amounts of protein were adsorbed on scaffolds loaded with nanosilicates (Fig. 3a). This might be attributed to higher surface roughness on scaffolds loaded with nanosilicates. Surface roughness is also expected to change the contact angle of water. However, we did not observe any significant effect of nanosilicates on the hydrophilic characteristics of PEOT/PBT (Fig. 3b); therefore, the enhanced protein adhesion on the scaffold may be attributed to the presence of nanosilicates. Earlier studies have shown that the electrostatic nature of nanosilicates can facilitate protein binding to a nanocomposite surface [36, 55].

Degradation characteristics of biomaterials play an important role in tissue regeneration, where scaffolds provide structural support to facilitate cell infiltration and the rate of new tissue regeneration matches that of scaffold degradation. Earlier studies have shown that PEOT/PBT degrade *via* hydrolysis and completely degrades over a period of 1 year under *in vivo* conditions [44]. By controlling the molar ratio of hard and soft segments within PEOT/PBT copolymer, a wide range of physical and degradation characteristics can be obtained [44]. The effect of the nanosilicate addition on degradation was determined by obtaining a cast PEOT/PBT film with and without nanosilicates. We used accelerated degradation conditions using 0.01 M NaOH to perform the degradation studies to expedite degradation rates and uncover trends between pure polymer and nanocomposite samples. Nanocomposite films evaluated under accelerated degradative conditions displayed higher stability and extended lifetimes with half-lives over three times longer than that of the pure polymer films (Fig. 3c). This suggested that interactions between nanosilicates and polymer chains protected PEOT/PBT against degradation *via* physical netpoint formation. Although these conditions provided an alternate degradative mechanism to those found *in vivo*, it provided insight as to the protective capabilities endowed by including nanosilicates. To understand the effect of degradation on scaffolds, surface morphology was evaluated after exposure to 0.01 M NaOH using SEM. Scaffolds composed of PEOT/PBT displayed smooth fiber morphology with signs of surface cracking and macro-scale degradation. Addition of nanosilicates to PEOT/PBT did not showed any sign of degradation and the surface was relatively smooth and the scaffold maintained the prefabricated architecture and profile. Although this base-catalyzed surface erosion mechanism was protected in nanocomposite structures, future studies are required to investigate long-term effects in aqueous environments, as hydrolytic degradation can modify degradation products. Based on previous studies investigating *in vivo* degradation of the copolymer hard and soft segments, long-term degradation products of 1000PEOT70PBT30 included PEO and terephthalate moieties [44]. Specifically, the insoluble products typically included PBT blocks, while soluble molecules were largely composed of PEG chains with small amounts of terephthalate (PEOT). From the perspective of nanosilicate-based composites, previous studies have indicated the pH buffering capabilities of the nanoparticles [56, 57]. Taken in combination, there is potential for these particles to reduce the potential of accelerated hydrolytic degradation *in vivo* and enhance stability as demonstrated here. Future studies are required to reach a conclusion, however, as the particles’ hydrophilic nature may promote more contact with water and increase degradation [49].

### Effect of nanosilicates on mechanical stability of 3D scaffolds

**Figure 4:**
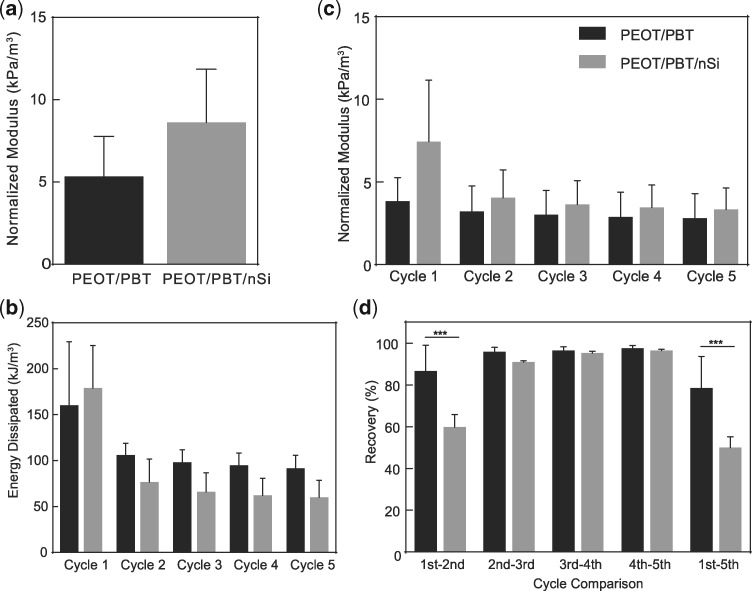
Mechanical characterization of 3D constructs. **(a)** The addition of nanosilicates had no negative impact on the compressive modulus of scaffolds. Addition of nanosilicates maintained scaffold architecture and resistance toward irreversible damage as indicated by no significant increases in **(b)** energy dissipation, **(c)** compressive modulus and **(d)** percentage recovery over the course of five compressive cycles

Mechanical stability of additive manufactured scaffolds is important to understand if they deform during and after implantation. It is likewise important that the scaffold withstands physiological loading and maintain structural integrity to facilitate tissue regeneration. PEOT is a hydrophilic polymer and will impart an elastomeric component, while PBT will provide stiffness to the copolymeric network as a more crystalline thermoplastic. Earlier studies have shown printed PEOT/PBT scaffolds can maintains structural integrity during *in vivo* implantation [21]. The effect of nanosilicates addition of PEOT/PBT scaffold was determined using uniaxial and cyclic compression testing. Volume occupied by polymer was accounted for during calculations due to size variations between pure polymer and nanocomposite structures. Specifically, the modulus of printed scaffold was normalized by fraction of volume occupied by printed scaffold. The results indicated no significant increase in mechanical stiffness of 3D-printed scaffold due to nanosilicate addition (Fig. 4a). Additionally, cyclic compression of scaffolds (40% strain) showed minimal breakdown indicating strong mechanical integrity of the printed scaffold over five cycles, as no change in compressive modulus was observed (Fig. 4b and c). The amounts of energy adsorbed and percentage recovery were calculated by loading and unloading cycles. Recovery analysis between cycles did indicate, however, a small initial deformation to the printed structure not noted in the pure polymer scaffolds (Fig. 4d). This phenomenon was not prevalent in the remaining cycles as the scaffold maintained stability of dynamic loading. Our results correlated well with previously reported study on mechanical properties of PEOT/PBT [9, 27]. Overall, these results indicated that the addition of nanosilicates maintained the mechanical strength and integrity of 3D scaffolds under cyclic compression, thereby providing physiological relevance for AM structures placed in dynamic environments.

### Effect of nanosilicates on hMSCs differentiation on 3D-printed scaffolds

**Figure 5:**
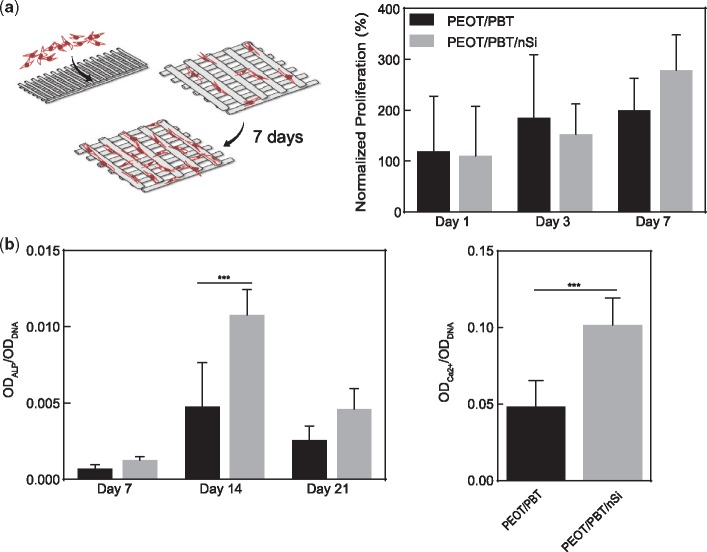
*In vitro* studies on bioactive nanocomposite scaffolds. **(a)** hMSCs seeded on 3D scaffolds proliferated over the course of the week. The effect of nanosilicate on hMSCs differentiation was evaluated by monitoring **(b)** ALP activity and **(c)** production of mineralized matrix. The presence of nanosilicates upregulate peak ALP activity (Day 14) and production of mineralized matrix (Day 21)

The initial stages of cell adhesion to the biomaterial surface are vital to the subsequent cellular events of spreading, proliferation and differentiation. To determine the effect of nanosilicates on hMSCs adhesion and differentiation on nanocomposites surface, hMSCs were seeded on scaffolds with and without nanosilicates. The proliferation of hMSCs was monitored using Alamar Blue assay. Earlier studies have shown that PEOT/PBT scaffolds supported hMSCs adhesion and proliferation; however, did not induce an osteogenic phenotype in hMSCs [58, 59]. Our results showed hMSCs viability and proliferation on both 3D scaffold compositions; however, no significant effect due to addition of nanosilicates was observed (Fig. 5a). The change in cell phenotype of seeded hMSCs was measured by evaluating activity of ALP, which is an early indicator of osteogenic differentiation. In undifferentiated hMSCs, ALP activity was low initially (∼1–7 days), while a peak was observed at 10–14 days to then reduce again at 21–24 days. Our results show a similar trend of ALP activity on 3D scaffolds. We also observed a significant increase in ALP activity on scaffolds containing nanosilicates (Fig. 5b). Temporal progression of hMSCs monitored *via* ALP activity indicates a successful initiation of hMSCs towards osteogenic lineages. Our results also showed that addition of nanosilicates also enhanced mineralized matrix production by hMSCs seeded on scaffolds (Fig. 5b), a more robust indicator of osteogenic phenotype induction. These results corroborate our earlier studies demonstrating the osteoinductive ability of nanosilicates [33, 35]. The presence of nanosilicates within the polymer scaffolds enables local dissolution of bioactive mineral ions from AM scaffolds, thereby stimulating stem cells towards an osteogenic phenotype. Earlier studies have also shown that the copolymer ratio (1000PEOT70PBT30) enable rapid calcification under *in vivo* conditions [19]. Based on current results and previous studies [19], it is expected that nanosilicates loaded PEOT/PBT scaffolds enhance bone regeneration and can be employed for bone tissue engineering.

Furthermore, this bioactivity of the printable nanocomposite addressed a primary shortcoming of current supportive polymeric scaffolds, like PCL or poly(D, L-lactic-co-glycolic acid) [60, 61]. Although filaments of these polymers provide structural integrity to constructs composed of the softer, water-swollen hydrogel bioinks, they lack bioactive residues that may improve and accelerate the formation of functional tissue with AM. The ability to control tissue formation beyond the hydrogel cell-carriers provides another tool for bioprinting researchers. Future *in vivo* studies will examine scaffold functionality with modifications to filament arrangement and macro structure.

## Conclusion

We successfully report synthesis and fabrication of bioactive scaffolds from PEOT/PBT and nanosilicate. Homogeneous distribution of nanosilicates within PEOT/PBT printed scaffolds was observed. The nanosilicate addition resulted in physiologically stable nanocomposites as nanosilicate reduced the degradation rate of PEOT/PBT. However, no significant increase in mechanical stiffness was observed. hMSCs readily proliferate on 3D scaffolds and inclusion of nanosilicates to the scaffolds resulted in significant upregulation of osteo-related proteins and production of mineralized matrix. Overall, our results showed that addition of nanosilicates to 3D PEOT/PBT scaffolds could provide a viable mechanism to induce bioactivity to the bone-binding polymer for bone tissue engineering.
